# Association of the Healthy Eating Index with Estimated Cardiovascular Age in Adults from the KNHANES 2013–2017

**DOI:** 10.3390/nu12102912

**Published:** 2020-09-23

**Authors:** Sunmin Park, Kyungjin Kim, Byung-Kook Lee, Jaeouk Ahn

**Affiliations:** 1 Department of Food and Nutrition, Obesity/Diabetes Center, Hoseo University, Asan 31499, Korea; smpark@hoseo.edu; 2 Graduate School of Medical Informatics, Soonchunhyang University, Asan 31538, Korea; spinedr@naver.com; 3 Department of Preventive Medicine, Soonchunhyang University, Asan 31538, Korea; bklee@sch.ac.kr; 4 Ibone Medical Center, Cheonan 31156, Korea

**Keywords:** cardiovascular disease, Korean Healthy Eating Index, fruit intake, fiber intake, vitamin C intake

## Abstract

In this paper, we hypothesized that the gap between estimated cardiovascular age (eCV-age) and chronological age had a gender-wise correlation with the Korean Healthy Eating Index (KHEI). We tested the hypothesis in adults aged 20–64 years old using the KNHANES 2013–2017 data. eCV-age was estimated based on the designated risk factors of cardiovascular disease (CVD) and age-gap was calculated by subtracting the eCV-age from the chronological age in 12,317 adults. Adjusted odds ratios for the age-gap were measured according to KHEI, while controlling for covariates to influence risk factors of CVD, using logistic regression analysis with the complex sample survey design. Age-gaps were divided into four groups: >4 (High), 0–4 (Moderate), −4–0 (Mild), and <−4 years (Low). The higher the age-gap, the lower the cardiovascular risk. Persons included in the following categories belonged to the high and moderate age-gap groups: young (<40 years), women, urban living, better than high school education, higher income, lean, mild drinking, and exercising regularly. KHEI scores were overall higher in women than men (*p* < 0.01). Having breakfast and saturated fat intake were primary factors that influenced the age-gap for men, whereas fresh fruit intake and carbohydrate intake influenced the age-gap in women. The KHEI scores positively correlated with nutrient intake, especially fiber and vitamin C intake in women (*p* < 0.05). Participants with high KHEI scores increased their chances of belonging to the high age-gap group by 2.16 times for men and 2.10 for women after adjusting for covariates of sex, age, and residence. However, after adding the covariates of education, income, marriage, and obesity, in conjunction with smoking, alcohol, and regular exercise, this reduced to 1.34 times in women. In conclusion, both genders had a positive correlation between age-gap and overall KHEI scores.

## 1. Introduction

Cardiovascular disease (CVD) is a collective term for disorders of the heart and blood vessels, including coronary heart disease, cerebrovascular disease, and heart failure, among others. CVD is the leading cause of death worldwide [1]. It is estimated that by 2030, about 23.6 million people will die worldwide from heart disease and CVD, including strokes [2]. In Korea, the mortality rate attributed to CVD has significantly decreased over the last 30 years. However, the mortality rate attributable to ischemic heart disease has shown a steady increase to date [3]. 

CVD can be prevented by reducing cardiovascular (CV) risk factors, which include hypertension, dyslipidemia, hyperglycemia, obesity, smoking, lack of a balanced diet, and physical exercise [4]. People with a greater number of risk factors are at a higher risk of developing CVD. The Korean National Health Insurance created a formula to estimate the cardiovascular age (eCV-age) of an individual, which represents the potential age when the person will develop CVD, calculated based on the risk factors [5]. If a person has multiple CV risk factors, the eCV-age will be higher than their chronological age. Modulating the risk factors can prevent and/or delay the incidence of CVD, thereby lowering the eCV-age. The eCV-age is thus a useful index to numerically represent the CV risk status of a person. The formula for eCV-age includes risk factors such as obesity, blood pressure, blood glucose, total cholesterol, glomerular filtration rate, proteinuria, smoking, and quantum of exercise, but does not include alcohol and nutrient intake although dietary patterns may also have a bearing on eCV-age [6]. 

It is well known that chronic diseases such as CVD can be influenced by both nutritional deficiencies and excessive nutrition [6]. Food and nutrient intakes from 24 h recall and semi-quantitative food frequency questionnaires (SQFFQ) and dietary patterns from the principal component analysis have been used to assess the association between nutrition, CVD, and metabolic syndrome [7]. It has, therefore, been suggested that eCV-age can be modified through alterations in eating habits. The Korea Ministry of Health and Welfare and the Korean Medical Association have established recommendations for optimal and balanced food, nutrient, and energy intake, which can help lower the eCV-age [8]. 

The Korea Centers for Disease Control and Prevention (KCDC) has developed the Korean Healthy Eating Index (KHEI) for adults, based on literature reviews, which established dietary guidelines for Korean adults. The 2010 Dietary Reference Intake for Koreans (2010 KDRI) outlined the relationship between obesity, abdominal obesity, metabolic syndrome, and nutrition [9]. KHEI is a good measure to assess the diet quality of Korean adults [9]. KHEI includes a total of 14 components: 8 items recommended for adequate food consumption, including breakfast, mixed grain, fruit, fruit juice, vegetables, fermented vegetables, meat and milk and milk products, three items for moderate consumption (i.e., saturated fatty acids, sodium, sweets, and beverages), and three items for balanced consumption (i.e., carbohydrates, total fat, and energy) [9]. A higher KHEI score indicates a healthy diet. In the Korea National Health and Nutrition Examination Survey (KNHANES VI) (2013–2015), the total score of KHEI in Korean adults was found to be 63.3, with the total score in men lower than that of women. Among the different age groups, the KHEI score was lowest in young adults between 20 and 40 years of age [9]. These KHEI scores reflect Korean dietary patterns and eating habits and their influence on eCV-age. The present study was based on the hypothesis that the gap between eCV-age and chronological age (age-gap) was associated with the Korean Healthy Eating Index (KHEI) score applied gender-wise to the Korean population. We tested the hypothesis in adults aged 20–64 years old using the KNHANES 2013–2017 data, a representative sample of the non-institutionalized civilian population.

## 2. Materials and Methods

### 2.1. Study Design and Data Collection

This study utilized data obtained from the Korea National Health and Nutrition Examination Survey (KNHANES) VI 2013–2017. KNHANES surveys are conducted annually using a rolling sampling design that involves a complex, stratified, multistage probability, and cluster survey of a representative sample of the non-institutionalized civilian population in South Korea. The survey was performed by the Korean Centers for Disease Control and Prevention (KCDC) and the Korean Ministry of Health and Welfare. A health interview, health examination, and nutrition survey were conducted. The survey was approved by the Institutional Review Board of the KCDC (approval no. 2013-07CON-03-4C). The study was conducted under the Helsinki Declaration (of 1975, as revised in 2008).

The cross-sectional analysis of KNHANES VI was restricted to 12,317 adults between 20–64 years of age who completed the health examination and nutrition survey. Detailed descriptions of the design of the survey have been previously reported [10]. Briefly, each participant’s age, residence area, education, income, smoking status, alcohol intake, and hours of physical activity were obtained during the health interview. Height and weight measurements were performed, with the participants wearing light clothing and no shoes. Body mass index (BMI) was calculated as weight in kilograms divided by the square of the height in meters (kg/m^2^). The age reported at the time of the health interview was categorized into five groups. The residence was categorized into urban and rural areas. Income level was categorized into four quartile groups, and education level was categorized into below high school, high school, college, or higher.

Alcohol consumption was assessed by asking the participants about their drinking behavior during the month before the interview, including average frequency (days per month) of alcoholic beverage consumption, and amount (in mL) of alcoholic beverages ingested on a single occasion. The responses were converted into the amount of pure alcohol (in grams) consumed per day. Drinking status was classified into none, mild (1–15) g, moderate (16–30) g, and heavy alcohol drinking (>30 g). Smoking status was defined based on self-reported cigarette use: “never-smokers” had smoked less than 100 cigarettes in their lifetime; participants who had smoked 100 or more cigarettes were considered “past or current smokers”, based on current cigarette use. Physical activity was defined as regular exercise ≥30 min at a time at least five times per week, as moderate exercise activities, or for ≥20 min at a time at least three times per week in vigorous exercise activities as described previously [10].

### 2.2. Definition of eCV-Age 

In Korea, all adults have a regular health check-up every two years, and the National Health Insurance Service has generated an estimation model of CV age using the data of the regular check-up according to the health appraisal logics [11]. CV age was estimated based on the risk factors and related treatment which influence CVD, including BMI, waist circumference, blood pressure, hypertension treatment, fasting blood glucose concentration, diabetes treatment, total serum cholesterol concentration, glomerular filtration rate, proteinuria detected by dipstick, smoking status, and exercise status [5,12]. Each risk factor was divided into (2 to 5) categories to give the odds ratio in each category in men and women (Table S1 of the Supplementary Information (SI)). The absolute risk values of each risk factor from Table S1 of the SI were added according to age and gender. The absolute risk value per person was multiplied by the average absolute risk of individual subjects for 10 years in Table S2 of the SI, according to age and gender. The average absolute risk of individual subjects (including and excluding total cholesterol measurement) was calculated in Korean adults who had regular check-ups for 10 years according to age and gender (Table S2 of the SI). The calculated person’s absolute risk was compared to the risk values in Table S3 of the SI. The eCV-age was designated by selecting the nearest absolute risk, and it was used as the estimated cardiovascular age of each subject. Age-gap was calculated by subtracting the eCV-age from the chronological age. The greater the age-gap, the lower the chance of having CVD. Age-gap scores were divided into three groups: >4 years (High), (0–4) (Moderate), (−4–0) (Mild), and <−4 years (Low).

### 2.3. Laboratory Testing

Blood samples were obtained in the morning, following an overnight fast. The serum concentrations of glucose, high-density lipoprotein cholesterol (HDL), triglycerides (TG), aspartate transaminase (AST), and alanine transaminase (ALT) were measured using a Hitachi automatic analyzer 7600 (Hitachi Ltd, Tokyo, Japan). Low-density lipoprotein cholesterol (LDL) was calculated using the Friedewald equation (LDL = total cholesterol − HDL − (TG/5)) if the TG concentration was not above 400 mg/dL. When the TG concentration was above 400 mg/dL, TG was measured directly using a Hitachi automatic analyzer 7600. All clinical analyses were performed by the Neodin Medical Institute, a laboratory certified by the Korean Ministry of Health and Welfare. 

### 2.4. Assessment of Food and Nutrient Intake from 24 h Recall and Food Frequency Questionnaires 

All subjects were instructed to maintain their usual dietary habits before the assessment of dietary intake. Daily nutrient intake was measured using the 24 h recall method. The 24 h recalls were conducted through in-person interviews by trained dieticians in mobile examination centers to explore food types and quantities that the subjects consumed for the previous 24 h (midnight to midnight) [10]. Daily intake of calories and nutrients was calculated from the information of food intake acquired from interviews using the Can-Pro 2.0 nutrient intake assessment software developed by the Korean Nutrition Society.

Dietary intake information was collected by administering a validated SQFFQ to each participant. The SQFFQ used in KNHANES was developed and validated by the Ministry of Health and Welfare (Sejong, Korea) [13]. This questionnaire requested information regarding the participant’s consumption of 113 food items. The participant’s food intake frequency was quantified using nine categories: never or seldom, once a month, two to three times a month, one to two times a week, three to four times a week, five to six times a week, once a day, twice a day, and three times or more every day.

### 2.5. KHEI Scores

KHEI is developed by KCDC to evaluate comprehensive diet quality among Koreans. It includes adequacy of food intake comprising 8 items, moderation of saturated fatty acids, sugar, and sodium intake, and balanced energy, fat, and carbohydrate intake [9,14]. KHEI scores were calculated based on KNHANES. The maximum scores for the items in the KHEI for adults aged 20–64 years were defined based on the Dietary Guidelines for Korean adults and dietary reference intakes for Koreans (2010) (Table S4). However, the recommendation criteria for sweets and beverage intake were adopted from the standards of the World Health Organization/ Food and Agriculture Organization of the United Nations, since these criteria have not yet been established for Koreans. 

### 2.6. Statistical Analysis

Statistical analyses were performed using SAS software (version 9.4; SAS Institute, Cary, NC, USA) and SUDAAN (Release 11.0; Research Triangle Institute, Research Triangle Park, NC, USA), a software package that incorporates sample weights and adjusted analyses for the complex sample survey design. Survey sample weights were used in all analyses to produce estimates that were representative of the non-institutionalized civilian Korean population. *p* values < 0.05 were considered as significant.

Descriptive statistics of participants according to age-gap groups with 4-year differences were obtained by determining the frequency distribution of categorical demographic variables, lifestyle factors, and the presence of metabolic syndrome components. Statistical significance was determined using chi-squared tests. 

Adjusted means and 95% confidence intervals (CI) of KHEI scores and macronutrient intake was calculated according to gender, and the eCV-age using multiple regression analysis after covariate adjustment. Covariates were sex, age, residence area, occupation, income, education level, marital status, drinking status, obesity, and physical activity.

Next, adjusted odds ratios (ORs) and 95% CI for having healthy eCV-age and its components according to the quartile score of KHEI were calculated using logistic regression analysis after covariate adjustment. Covariates were sex, age, residence area, occupation, income, education level, marital status, drinking status, obesity, and physical activity. 

## 3. Results

### 3.1. Socioeconomic and Lifestyle Characteristics of the Participants According to the Age-Gap Groups 

The distribution of socioeconomic and lifestyle characteristics showed significant variation among the age-gap groups (Table 1; *p* < 0.01). There was a gender difference among the age-gap groups: the highest number of women belonged in the (0–4) year group, but the highest number of men belonged in the (−4–0) year group. The highest number of the elderly belonged in the highest and lowest age-gap groups as compared to the young adults (Table 1). The highest number of persons living in urban areas belonged in the (0–4) year group compared with those in rural areas. More persons with higher-education and higher-income belonged to the >4-year and (0–4) year groups than those with lower-education and lower-income (Table 1). Drinking status showed that none and mild drinkers were highest in the >4-year and (0–4) year eCV-age groups and that the persons with heavy drinking were highest in the (−4–0) year group. Exercise status significantly but minimally affected the estimated cardiovascular age: the persons with exercise were higher in the >4 and (0–4) year groups than those with no-exercise, but the differences were not substantial (Table 1). The combined percentage of people in the highest and high age-gap groups was about 50% in all years and the percentage in 2016 was marginally lower than that in 2013.

### 3.2. KHEI Scores for Each Gender According to the Age-Gap Groups

In those consuming “adequate” items, among men, those having breakfast were in the higher age-gap group, with higher scores. (Table 2). However, in women, the scores of those having only fresh fruit intake were significantly different among the age-gap groups. These results indicate that men having breakfast and women consuming higher fresh fruits had lower eCV-age, compared to their chronological age. 

In those consuming “moderate” items, the percentage of energy from saturated fatty acids showed a significant variation among the age-gap groups only in men (Table 2). Men consuming lower saturated fatty acids as a proportion of daily energy intake had lower eCV-age, compared to chronological age. However, women did not exhibit any significant variation in this group. There was no significant variation among the age-gap groups in the category of the balance of energy intake in men. However, in women, carbohydrate intake (% energy) was significantly different among the age-gap groups, and women in the >4-year group showed significantly lower intake of carbohydrates (Table 2). 

Women had higher total KHEI scores than men. Men did not show any significant variation among the age-gap groups, but women had significantly higher values of total KHEI in the >4-year and (0–4) year groups than in the other age-gap groups (Table 2). Therefore, women with the highest KHEI scores had lower eCV-age than their chronological age.

### 3.3. Nutrient Intake Calculated from 24 h Recall

Men and women showed a similar pattern of daily nutrient intake calculated by 24 h recall. Interestingly, fiber intake was higher in the >4-year age-gap group than the other groups in both genders (Table 3). However, vitamin C intake was higher in the >4-year age-gap group than the other groups only in women. Other nutrient and energy intakes were not significantly different among the age-gap groups (Table 3).

### 3.4. Association of KHEI with a Healthy eCV-Age

Reference of the logistic regression analysis was the highest KHEI score group, indicating that the reference was the best quality of diet intake. In model 1, the adjusted odds ratio for having healthy eCV-age had a positive association with the highest KHEI by (2.16 and 2.10) times in men and women, respectively, after adjusting for sex, age, residence, and region (Table 4). It indicated that the person with the highest KHEI had a higher chance to have the lowest eCV-age, compared to chronological age. In model 2, the adjusted odds ratio for having healthy eCV-age also showed a positive association with the highest KHEI by 1.79 times for men and 2.38 times for women, from the lowest KHEI after adjusting covariates for model 1 plus education, income, marriage, and obesity (Table 4). However, in model 3, adjusted for model 2 plus smoking, alcohol, and regular exercise, the OR for having healthy eCV-age was positively associated with KHEI by 1.34 times only in women, and there was no significant association between healthy eCV-age and KHEI in men (Table 4). 

## 4. Discussion

The current study validates the hypothesis that the gap between eCV-age and chronological age (age-gap) based on the KHEI scores applied gender-wise to a representative sample of the non-institutionalized Korean civilian population, aged 20–64 years old using the KNHANES 2013–2017 data is a modifiable nutrition-based factor. To the best of our knowledge, this the first study to show a correlation between age-gap and KHEI scores.

Previous reports have shown that eCV-age is an indicator of susceptibility to CVD. An increase in age-gap could lead to a reduction in mortality associated with cardiovascular diseases. In the current study, participants with a higher age-gap had the following characteristics: female, <50 years old, urban living, ≥high school education, higher income, non-smoker, non- or mild drinker, and regular exercisers. These results were consistent with previous studies [15,16]. What this study adds is that appropriate meal management could also be an additional factor to increase the age-gap, which in turn could lower the risk of CVD and related mortality [17,18]. 

Thus, far eCV-age estimation has not included food intake patterns. There are several methods to evaluate the person’s food intake using SQFFQ and the 24 h recall method. The KCDC constructed the KHEI scores to evaluate Korean food intake and includes the adequacy of beneficial food intake, moderation of harmful food intake, and balance of energy intake [8,9]. The total scores of KHEI were higher in women (about 64.0) than men (about 58.5) in the present study. These results indicated that women had better meal management than men. The KHEI results were consistent with those of eCV-age: a higher proportion of women (55.3%) belonged to the highest and high age-gap groups than did men (44.9%). The Alternative Healthy Eating Index (AHEI-2010) derived using FFQs is similar to KHEI to evaluate diet quality [19]. 

Interestingly, in this study, the total scores of KHEI were significantly associated with the eCV-age differences only in women after adjusting all covariates, but not men. The eCV-age in men was mediated more by smoking, alcohol drinking, and regular exercise than eating habits. Consistent with the present study, many studies have shown that higher scores of AHEI are associated with favorable concentrations of cardiometabolic and endocrine biomarkers, including inflammation index [20,21]. In the Women’s Lifestyle Validation Study, different diet patterns, including the Dietary Approaches to Stop Hypertension diet, alternative Mediterranean diet, and Alternative Healthy Eating Index (AHEI), were found to be significantly associated with the biomarkers related to type 2 diabetes and the incidence of CVD in a cross-sectional analysis of 775 healthy women [20]. The statistical significance of the association increased after adjustment of BMI, indicating that BMI partly mediates the association. These results suggest that the compliance of KHEI-recommended dietary patterns might be involved in reducing the incidence of CVD.

Carbohydrate, protein, and fat intake calculated from the 24 h recall data were not significantly different among the age-gap groups using the data of KNHANES 2013–2017 in the present study. However, previous studies have demonstrated that a very low-fat diet and a high carbohydrate diet elevates the risk of metabolic syndrome in the KNHANES 2007–2012 [10,22]. Their ratios in the KNHANES 2013–2017 were, however, different from those in the KNHANES 2007–2012 (carbohydrate: protein: fat = 72.0:13.4:13.7 energy percent) [10,22]. While fat and protein intake has increased, recently it has not had an impact on the incidence of metabolic syndrome and CVD related mortality. Interestingly, the scores of fresh fruit intake in KHEI were significantly and positively associated with age-gap groups in both genders in the present study. Consistent with the KHEI scores for fresh fruits intake rich in fiber and vitamin C, fiber intake was highest in the highest age-gap group among all the groups in both genders. However, vitamin C intake was highest among the age-gap groups only in women, not men. Consistent with the present study, the consumption of foods rich in dietary fiber, including fruits, vegetables, and the cereal has been shown to lower the risk of metabolic syndrome in a prospective study and a randomized controlled trial (RCT) [23,24]. However, in the National Health and Nutrition Examination (NHANES) 2013–2016 survey, 100% fruit juice was associated with the improvement of diet quality [25]. In our study, however, fruit intake, but not fruit juice, enhanced diet quality, specifically in women.

This study was conducted using a rolling sampling design that involved a complex, stratified, multistage probability cluster survey designed to represent the non-institutionalized civilian population in South Korea. The study had some limitations. First, the results could not be interpreted as cause-and-effect because this study was a cross-sectional study. Second, although SQFFQ included 116 items that Koreans have usually consumed, some foods were not included in the SQFFQ, which in turn could have affected the results. A 1-day 24-h dietary recall might not reflect the usual daily nutrient intake. However, 24 h recall was conducted by a skilled technician, thereby reducing measurement errors. Finally, the estimation model for CVD might not estimate the eCV-age correctly, since the model included total serum cholesterol concentrations, but not serum triglyceride, LDL, and HDL concentrations [10,26]. CVD is known to be associated with low serum HDL and high LDL cholesterol and triglyceride concentrations. Total serum cholesterol concentrations may not be an accurate indicator of dyslipidemia status. 

## 5. Conclusions

We accept the hypothesis that both genders had a positive association with age-gap in overall KHEI scores, but there were gender-wise differences in the individual components of KHEI and age-gap. Having breakfast and saturated fat intake were primary factors to influence the age-gap for men, whereas fresh fruit intake and carbohydrate intake influenced the age-gap in women. The KHEI scores calculated from SQFFQ positively correlated with nutrient intake, especially fiber and vitamin C intake from 24 h recall in women. These results suggest that the association of KHEI scores with age-gap would be well-represented in Korean adults. However, the eCV-age model may need to be modulated for dyslipidemia, a better index of the e-CV-age.

## Figures and Tables

**Table 1:** Distribution of study population according to the age-gap groups.

Classification Variables		Age-Gap Groups ^1^				
		>4 ^2^ (*N* = 1252)	0–4 (*N* = 5000)	−4–0 (*N* = 4448)	<−4.0 (*N* = 1617)	*p* Value ^3^
Sex	Male	493 (9.1)	1693 (35.8)	2039 (42.5)	661 (12.6)	<0.01
	Female	759 (9.4)	3307 (45.8)	2409 (31.8)	956 (12.9)	
Age group	20–29	0 (0)	866 (48)	748 (43.4)	165 (8.6)	<0.01
	30–39	71 (3.1)	1495 (50.8)	1041 (37.1)	253 (8.9)	
	40–49	375 (12.3)	1202 (37.8)	1039 (34.4)	474 (15.4)	
	50–59	554 (17.6)	996 (31.6)	1119 (34.6)	482 (16.1)	
	60–64	252 (17.5)	441 (30.5)	501 (35.3)	243 (16.7)	
Residence	Urban	1043 (9.3)	4256 (42)	3654 (36.5)	1292 (12.3)	<0.01
	Rural	209 (9.3)	744 (35.5)	794 (39.9)	325 (15.3)	
Education	<high school	715 (11.6)	1887 (32.8)	2196 (39)	970 (16.6)	<0.01
	High school	125 (5.6)	889 (45.5)	703 (37.6)	215 (11.2)	
	College	412 (8.3)	2224 (47.8)	1549 (34.6)	432 (9.3)	
Income	1st Q	100 (7.9)	335 (31.6)	443 (41.6)	228 (18.9)	<0.01
	2nd Q	276 (8.3)	1124 (38.7)	1134 (38.6)	437 (14.3)	
	3rd Q	365 (8.4)	1652 (42.5)	1424 (37.3)	482 (11.8)	
	4th Q	509 (11.1)	1875 (43.5)	1434 (34.5)	461 (10.9)	
Drinking status	None	424 (13.8)	1187 (39.9)	984 (33.4)	392 (13)	<0.01
	Mild	699 (9.7)	2990 (46.3)	2195 (33.7)	734 (10.3)	
	Moderate	87 (5.8)	474 (35.3)	609 (44.7)	190 (14.1)	
	Severe	42 (2.7)	349 (25.7)	660 (49.9)	301 (21.7)	
Smoking status	Non-smoker	1078 (13.6)	3754 (50.7)	2090 (25.6)	807 (10.1)	<0.01
	Past smoker	174 (7.7)	729 (37.7)	876 (42)	277 (12.7)	
	Current smoker	0 (0)	517 (20.1)	1482 (60.6)	533 (19.3)	
Exercise	Yes	654 (9.7)	2498 (41.8)	2177 (36.7)	731 (11.7)	<0.01
	No	598 (8.8)	2502 (40.1)	2271 (37.3)	886 (13.8)	
Year	2013	308 (8.8)	1380 (42.7)	1164 (36.1)	409 (12.4)	<0.01
	2014	330 (10.1)	1239 (41.7)	1041 (36)	370 (12.2)	
	2015	335 (10.1)	1141 (40.4)	1104 (37.7)	371 (11.8)	
	2016	279 (8.1)	1240 (39.1)	1139 (38.1)	467 (14.6)	

^1^ The age-gap calculated by subtracting the chronological age to estimated cardiovascular age (eCV-age). The bigger values of age-gap indicated less risk of cardiovascular diseases than the chronological age. ^2^ As the bigger age-gap, the lower the chance to have cardiovascular diseases. The age-gap was categorized into >4 years, 0–4 years, −4–0 years, and <−4 years. ^3^ Chi-square test for each classification variable for eCV-age groups.

**Table 2:** Adjusted ^1^ means and 95% confidence intervals of the Korean Healthy Eating Index Score (KHEI) by the age-gap ^2^ groups.

Classification		Male				Female			
		>4 Years (*N* = 493)	0–4 Years (*N* = 1693)	−4–0 Years (*N* = 2039)	<−4 Years (*N* = 661)	>4 Years (*N* = 759)	0–4 Years (*N* = 3307)	−4–0 Years (*N* = 2409)	<−4 Years (*N* = 956)
Adequacy	Have breakfast	6.91 (6.53–7.29)	6.96 (6.76–7.17)	6.48 (6.28–6.68)	6.67 (6.32–7.02) *	7.34 (7.05–7.62)	7.03 (6.86–7.19)	6.93 (6.73–7.12)	6.96 (6.66–7.25)
	Mixed grains intake	3.74 (3.53–3.95)	3.76 (3.64–3.87)	3.8 (3.7–3.9)	3.85 (3.67–4.03)	4.11 (3.98–4.25)	4.16 (4.09–4.23)	4.07 (3.98–4.15)	4.17 (4.03–4.31)
	Total fruits intake	2.42 (2.21–2.62)	2.47 (2.36–2.58)	2.52 (2.42–2.62)	2.33 (2.16–2.49)	3.92 (3.8–4.04)	3.79 (3.72–3.86)	3.73 (3.65–3.81)	3.73 (3.6–3.85)
	Fresh fruits intake	2.21 (2.01–2.42)	2.2 (2.09–2.31)	2.25 (2.16–2.35)	2.03 (1.88–2.18)	3.83 (3.71–3.96)	3.69 (3.63–3.76)	3.6 (3.51–3.68)	3.63 (3.51–3.76) *
	Total vegetable intake	4.84 (4.77–4.9)	4.78 (4.73–4.82)	4.8 (4.76–4.83)	4.78 (4.72–4.85)	4.87 (4.83–4.9)	4.88 (4.86–4.91)	4.86 (4.83–4.89)	4.87 (4.82–4.92)
	Vegetable intake excluding kimchi and pickled vegetables	3.81 (3.64–3.98)	3.78 (3.68–3.87)	3.77 (3.69–3.84)	3.8 (3.65–3.95)	4.32 (4.22–4.41)	4.35 (4.3–4.4)	4.3 (4.24–4.36)	4.29 (4.18–4.39)
	Meat, fish, eggs, and beans intake	3.97 (3.79–4.14)	3.94 (3.86–4.02)	3.94 (3.87–4.02)	3.93 (3.78–4.08)	4.07 (3.89–4.25)	4.02 (3.94–4.1)	3.99 (3.9–4.08)	3.97 (3.83–4.11)
	Milk and milk products intake	2.76 (2.29–3.22)	3.32 (3.09–3.55)	3.52 (3.3–3.74)	3.36 (2.98–3.75)	3.92 (3.56–4.27)	3.77 (3.57–3.96)	3.57 (3.37–3.77)	3.46 (3.16–3.77)
	Total scores of the adequacy	30.6 (29.7–31.6)	31.2 (30.6–31.7)	31.1 (30.6–31.6)	30.8 (30.0–31.6)	36.4 (35.7–37.1)	35.7 (35.3–36.1)	35.0 (34.6–35.5)	35.1 (34.4–35.8) *
Moderation	% of energy from saturated fatty acids	9.62 (9.53–9.72)	9.43 (9.37–9.5)	9.43 (9.38–9.49)	9.48 (9.39–9.58) *	9.44 (9.36–9.51)	9.41 (9.36–9.46)	9.41 (9.35–9.46)	9.36 (9.27–9.45)
	Sodium intake	4.13 (3.71–4.55)	3.86 (3.65–4.07)	3.84 (3.66–4.02)	3.62 (3.32–3.92)	5.37 (5.06–5.68)	5.28 (5.13–5.43)	5.28 (5.11–5.45)	5.04 (4.74–5.34)
	% of energy from sweets and beverage	3.93 (3.72–4.14)	3.94 (3.82–4.05)	4.05 (3.95–4.15)	4.00 (3.81–4.18)	3.78 (3.61–3.95)	3.57 (3.49–3.66)	3.68 (3.59–3.78)	3.73 (3.57–3.89)
	Total scores of the moderation	17.6 (17.1–18.1)	17.2 (16.9–17.4)	17.3 (17.0–17.5)	17.0 (16.6–17.4)	18.4 (18.1–18.8)	18.2 (18.0–18.4)	18.3 (18.0–18.5)	18.0 (17.6–18.4)
Balance of energy intake	% of energy from CHO	3.02 (2.8–3.23)	3.31 (3.2–3.42)	3.31 (3.22–3.41)	3.2 (3.03–3.38)	3.01 (2.85–3.17)	3.26 (3.19–3.34)	3.19 (3.11–3.28)	3.15 (3–3.3) *
	% of energy from fat	3.97 (3.79–4.14)	4.03 (3.95–4.11)	4.03 (3.96–4.1)	3.95 (3.81–4.09)	4.06 (3.91–4.21)	4.16 (4.1–4.21)	4.14 (4.08–4.21)	4.04 (3.93–4.15)
	Energy intake	3.91 (3.71–4.11)	3.99 (3.88–4.09)	3.91 (3.81–4)	3.95 (3.77–4.12)	3.9 (3.75–4.04)	3.94 (3.87–4.02)	3.95 (3.86–4.03)	3.99 (3.85–4.13)
	Total scores of the energy intake	10.0 (9.61–10.5)	10.5 (10.3–10.7)	10.5 (10.3–10.6)	10.3 (10.0–10.7)	10.1 (9.79–10.5)	10.5 (10.3–10.6)	10.4 (10.2–10.6)	10.4 (10.2–10.7)
Total scores of KHEI		58.3 (57.3–59.3)	58.9 (58.4–59.4)	58.8 (58.3–59.3)	58.1 (57.3–58.9)	64.9 (64.2–65.7)	64.3 (63.9–64.7)	63.7 (63.2–64.2)	63.5 (62.7–64.3) *

^1^ Adjusted by age, residence, region, education, obesity, income, drinking status, smoking status, marriage, and exercise. CHO, carbohydrates. ^2^ The age-gap calculated by subtracting the chronological age to estimated cardiovascular age (eCV-age). A higher value indicated a better dietary state of each item. * Significantly different among the eCV-age groups at *p* < 0.05.

**Table 3:** Adjusted mean ^1^ and 95% confidence intervals of major nutrient intake calculated from 24-h recall method according to genders and the age-gap groups ^2^.

	Male				Female			
	>4 Years (*N* = 493)	0–4 Years (*N* = 1693)	−4–0 Years (*N* = 2039)	<−4.0 Years (*N* = 661)	>4 Years (*N* = 759)	0–4 Years (*N* = 3307)	−4–0 Years (*N* = 2409)	<−4 Years (*N* = 956)
Energy (kcal/day)	2509 (2400–2618)	2522 (2465–2580)	2589 (2534–2644)	2643 (2551–2735)	1854 (1797–1910)	1786 (1756–1816)	1802 (1767–1837)	1800 (1740–1860)
Fat (En%)	20.6 (19.8–21.5)	20.8 (20.3–21.3)	20.5 (20.1–20.9)	20.4 (19.6–21.2)	20.9 (20.1–21.6)	20.8 (20.4–21.1)	20.6 (20.2–21.0)	19.9 (19.3–20.6)
Carbohydrate (En%)	59.1 (57.8–60.4)	58.1 (57.4–58.9)	58.0 (57.3–58.7)	56.6 (55.3–57.9)	63.3 (62.3–64.3)	63.2 (62.7–63.6)	63.1 (62.6–63.6)	63.2 (62.3–64.1)
Protein (En%)	14.4 (13.9–14.8)	14.2 (14.0–14.4)	14.0 (13.9–14.2)	14.1 (13.8–14.5)	14.1 (13.7–14.4)	14.0 (13.8–14.2)	14.0 (13.8–14.2)	14.0 (13.6–14.3)
Fiber (g/day)	27.3 (25.7–29.0)	25.5 (24.8–26.3)	26.6 (25.9–27.3)	26.7 (25.6–27.8) *	23.7 (22.6–24.9)	22.3 (21.8–22.8)	22.1 (21.5–22.6)	21.8 (20.9–22.8) *
Ca (mg/day)	571 (533–610)	579 (559–599)	568 (552–585)	572 (544–599)	472 (452–493)	457 (446–468)	447 (436–459)	444 (423–465)
Fe (mg/day)	20.1 (18.0–22.3)	21.2 (18.4–24.0)	21.3 (19.7–22.8)	21.3 (18.4–24.1)	16.2 (15.5–17.0)	15.6 (15.2–15.9)	15.5 (15.0–15.9)	14.9 (14.2–15.6)
Vitamin C (mg/day)	103 (87.3–119)	99.1 (92.9–105)	98.8 (92.9–105)	101 (90.6–112)	128 (113–143)	108 (103–114)	107 (101–113)	102 (94.2–111) **

^1^ Adjusted by age, residence, region, education, obesity, income, drinking status, smoking status, marriage, and exercise. ^2^ The age-gap calculated by subtracting the chronological age to estimated cardiovascular age (eCV-age). En%, energy percentage. * Significantly different among the eCV-age groups by Chi-square test at *p* < 0.05 and ** at *p* < 0.01.

**Table 4:** Adjusted odds ratios (95% confidence intervals) for having healthy estimated cardiovascular age (cardiovascular age < chronological age) after adjustments for covariates.

Korean Healthy Eating Index (KHEI)		Male	Female	All
Model 1	Q1	Reference (1.000)	Reference (1.000)	Reference (1.000)
	Q2	1.338 (0.982–1.823)	1.429 (1.204–1.696)	1.363 (1.184–1.57)
	Q3	1.637 (1.197–2.24)	1.755 (1.498–2.056)	1.68 (1.465–1.926)
	Q4	2.155 (1.576–2.947)	2.103 (1.811–2.443)	2.087 (1.826–2.384)
Model 2	Q1	Reference (1.000)	Reference (1.000)	Reference (1.000)
	Q2	1.424 (1.192–1.702)	1.537 (1.104–2.14)	1.4 (1.204–1.628)
	Q3	1.575 (1.331–1.865)	1.888 (1.356–2.63)	1.637 (1.417–1.891)
	Q4	1.788 (1.514–2.111)	2.384 (1.715–3.313)	1.949 (1.686–2.254)
Model 3	Q1	Reference (1.000)	Reference (1.000)	Reference (1.000)
	Q2	1.312 (0.915–1.883)	1.234 (1.017–1.497)	1.265 (1.073–1.49)
	Q3	1.222 (0.853–1.752)	1.248 (1.036–1.504)	1.215 (1.039–1.42)
	Q4	1.273 (0.887–1.828)	1.339 (1.109–1.617)	1.27 (1.086–1.487)

Model 1: adjusted for sex, age, residence, and region. Model 2: adjusted for model 1 plus education, income, marriage, and obesity. Model 3: adjusted for model 2 plus smoking, alcohol, regular exercise. Q1, Q2, Q3 and Q4 indicated the lowest, low, high and highest values of KHEI by dividing KHEI into the quartiles, respectively.

## Supplementary Materials

The following are available online at https://www.mdpi.com/2072-6643/12/10/2912/s1, Table S1: Logic of health risk appraisal for cardiovascular disease, Table S2: Average absolute risk (10 years) of individual subjects according to the type of health examination (including and excluding total cholesterol measurement), Table S3: Absolute risk (10 years) of individual subject by age and genders, Table S4: Components of and scoring standards for Korean Healthy Eating Index.
