# Supplementary material for: Association of the Healthy Eating Index with Estimated Cardiovascular Age in Adults from the KNHANES 2013–2017
Source: Nutrients. 2020 Sep 23;12(10):2912. doi: 10.3390/nu12102912 (PMC7598165; doi:10.3390/nu12102912)
Supplement: Supplementary file 1 [file nutrients-12-02912-s001.pdf]

Supplementary Table 1. Logic of health risk appraisal for cardiovascular disease

Risk Factors	Criteria	Risk (Male)	Risk (Female)	Remark
BMI (kg/m^2)	< 25.0	1	1	Select the higher risk between BMI and WC
	25.0 – 26.4	1.04	1.02	
	26.5 – 27.9	1.15	1.03	
	28.0 – 29.9	1.36	1.15	
	≥30.0	1.58	1.33	
Waist circumference (cm)	<90 (Male)/85 (Female)	1	1	
	≥90 (Male)/ 85 (Female)	1.44	1.52	
Blood pressure (mmHg)	<120 and <80	1	1	
	120-139 or 80-89	1.25	1.43	
	140-159 or 90-99	1.78	2.06	
	≥160 or ≥100	2.71	3.2	
Treatment of hypertension	No	1	1	
	Yes	1.22	1.22	
Fasting blood sugar (mg/dL)	<100	1	1	
	100-109	1.04	1.03	
	110-125	1.12	1.14	
	126-139	1.27	1.31	
	≥140	1.75	1.8	
Treatment of diabetes	No	1	1	
	Yes	1.42	1.42	
Total cholesterol (mg/dL)	<200	1	1	
	200-239	1.15	1.07	
	≥240	1.37	1.2	
GFR (MDRD) (ml/min/1.73m2)	≥60	1	1	Select the higher risk between GFR and Dipstick proteinuria
	≥45 &<60	1.34	1.34	
	<45	2.09	2.09	
Proteinuria by dipstick	None/trace/1+	1	1	
	2+	1.69	1.69	
	3+ or above	2.43	2.43	
Smoking status	None smoking	1	1	
	Past smoking	1.3	1.2	
	Current smoking	1.6	1.6	
Exercise	≥3 days per week	1	1	
	≤2 days per week	1.2	1.2	

Supplementary Table 2. Average absolute risk (10 years) of individual subjects according to the type of health examination (including and excluding total cholesterol measurement)

Age	Including Total Cholesterol		Excluding Total Cholesterol	
	Male	Female	Male	Female
20-24	1.83	1.43	1.8	1.42
25-29	1.91	1.4	1.86	1.38
30-34	2.02	1.44	1.94	1.41
35-39	2.08	1.47	1.98	1.45
40-44	2.11	1.54	2.01	1.51
45-49	2.14	1.64	2.04	1.6
50-54	2.19	1.78	2.09	1.72
55-59	2.23	1.92	2.14	1.85
60-64	2.25	2.08	2.17	2.02
65-69	2.28	2.22	2.2	2.16
70-74	2.25	2.32	2.18	2.26
75-	2.24	2.4	2.18	2.35

Table 3. Absolute risk (10 years) of individual subject by age and genders

Age	Absolute Risk		Age	Absolute Risk	
	Male	Female		Male	Female
20	99	92	53	5,573	2,905
21	138	103	54	5,988	3,201
22	178	115	55	6,403	3,496
23	217	126	56	6,818	3,792
24	256	137	57	7,234	4,088
25	295	149	58	7,928	4,720
26	334	160	59	8,622	5,353
27	374	172	60	9,316	5,986
28	449	211	61	10,010	6,619
29	524	250	62	10,705	7,252
30	600	289	63	11,462	8,001
31	675	328	64	12,219	8,750
32	751	368	65	12,976	9,499
33	873	402	66	13,733	10,248
34	995	437	67	14,491	10,997
35	1,117	472	68	15,205	11,975
36	1,239	507	69	15,919	12,953
37	1,362	542	70	16,633	13,931
38	1,557	646	71	17,347	14,909
39	1,752	750	72	18,062	15,887
40	1,947	854	73	18,380	16,307
41	2,142	958	74	18,698	16,728
42	2,337	1,062	75	19,017	17,148
43	2,591	1,143	76	19,335	17,569
44	2,846	1,225	77	19,654	17,990
45	3,101	1,307	78	19,972	18,410
46	3,356	1,389	79	20,290	18,831
47	3,611	1,471	80	20,609	19,251
48	3,920	1,698	81	20,927	19,672
49	4,229	1,926	82	21,246	20,093
50	4,539	2,154	83	21,564	20,513
51	4,848	2,382	84	21,882	20,934
52	5,158	2,610	85	22,201	21,354

Supplementary Table 4. Components of and scoring standards for Korean Healthy Eating Index

Components (score range)	Reference for score of each component	
	Maximum score	Minimum score
Having breakfast (0–10)	5–7 day/week	0 day/week
Whole grain (0–5)	≥0.3 serving/day	0 serving/day
Total fruit, including juice (0–5)	Men: (aged 19–64) ≥3 serving/day, (aged≥65) ≥2 serving/day<br>Women: (aged 19–64) ≥2 serving/day, (aged≥65) ≥1 serving/day	0 serving/day
Fruit, excluding juice (0–5)	Men: (aged 19–64) ≥1.5 serving/day, (aged≥65) ≥1 serving/day<br>Women: (aged 19–64) ≥1 serving/day, (aged≥65) ≥0.5 serving/day	0 serving/day
Total vegetable, including Kimchi and pickles (0–5)	Men: ≥8 serving/day<br>Women: (aged 19–64) ≥8 serving/day, (aged≥65) ≥6 serving/day	0 serving/day
Vegetable, excluding Kimchi and pickles (0–5)	Men: ≥5 serving/day<br>Women: (aged 19–64) ≥5 serving/day, (aged≥65) ≥3 serving/day	0 serving/day
Meat, fish, eggs, and legumes (0–10)	Men: (aged 19–64) ≥5 serving/day, (aged≥65): ≥4 serving/day<br>Women: (aged 19–64) ≥4 serving/day, (aged≥65) ≥2.5 serving/day	0 serving/day
Milk and dairy (0–10)	≥1 serving/day	0 serving/day
Sodium (0–10)	≤2,000 mg/day	>6,500 mg/day
Saturated fatty acid (0–10)	≤7% of energy	>10 % of energy
Empty calorie foods (0–10)	≤10% of energy	>20 % of energy
Carbohydrate (0–5)	55–65% of energy	<50%, >75% of energy
Fat (0–5)	15–30% of energy	<10%, >35% of energy
Total energy (0–5)	75–125% of EER by sex and age group	<60%, >140% of EAR

EER = estimated energy requirement.
